# Our Journal

**Published:** 1867-09

**Authors:** 


### OUR JOURNAL.

We have concluded arrangements which will insure foreign correspondence for some time to come. These letters will be very interesting, doubtless, as they will be the result of "sight-seeing" in the medical emporiums of the world, London and Paris.

We expect a letter, at least once a month, during the fall and winter, and hope to have one from each of these great centres of medical learning, in every number of the Journal, for some time to come.

In the mean time, it will be our object, constantly, to afford our readers all that is new and interesting in Medicine and Surgery, from all the various sources at our command.

In this connection, we would not pass unnoticed the contributions of practicing physicians in our own country. These, to us, and no doubt to our readers, are both interesting and instructive. That is most beautiful which is most useful; and the inventions and discoveries of industrious, practical physicians, directed by science, are important additions to the medical literature of the Country. The knowledge of many of such facts die with their discoverer, on account of the disinclination of practitioners to make contributions to periodical.

We, therefore, persuade and encourage physicians in country practice—where, from the inability to obtain known contrivances and remedies, they are often driven to important discoveries—to make known, through medical Journals, such useful information.

